# Multi-functional bismuth-doped bioglasses: combining bioactivity and photothermal response for bone tumor treatment and tissue repair

**DOI:** 10.1038/s41377-018-0007-z

**Published:** 2018-05-18

**Authors:** Liping Wang, Nicholas J. Long, Lihua Li, Yao Lu, Mei Li, Jiangkun Cao, Yu Zhang, Qinyuan Zhang, Shanhui Xu, Zhongmin Yang, Chuanbin Mao, Mingying Peng

**Affiliations:** 1 The State Key Laboratory of Luminescent Materials and Devices, Guangdong Engineering Technology Research and Development Center of Special Optical Fiber Materials and Devices, Guangdong Provincial Key Laboratory of Fiber Laser Materials and Applied Techniques, School of Materials Science and Engineering, South China University of Technology, 510641 Guangzhou, China; 2 Department of Chemistry, Imperial College London, South Kensington, London, SW7 2AZ UK; 3 Guangdong Key Lab of Orthopedic Technology and Implant Materials, Department of Orthopedics, Guangzhou General Hospital of Guangzhou Military Command, 111 Liuhua Road, 510010 Guangzhou, China; 4 Department of Chemistry and Biochemistry Stephenson Life Sciences Research Center, University of Oklahoma, Norman, OK 73072 USA; 5 School of Materials Science and Engineering, South China University of Technology, Guangzhou, 510641 China

## Abstract

Treatment of large bone defects derived from bone tumor surgery is typically performed in multiple separate operations, such as hyperthermia to extinguish residual malignant cells or implanting bioactive materials to initiate apatite remineralization for tissue repair; it is very challenging to combine these functions into a material. Herein, we report the first photothermal (PT) effect in bismuth (Bi)-doped glasses. On the basis of this discovery, we have developed a new type of Bi-doped bioactive glass that integrates both functions, thus reducing the number of treatment cycles. We demonstrate that Bi-doped bioglasses (BGs) provide high PT efficiency, potentially facilitating photoinduced hyperthermia and bioactivity to allow bone tissue remineralization. The PT effect of Bi-doped BGs can be effectively controlled by managing radiative and non-radiative processes of the active Bi species by quenching photoluminescence (PL) or depolymerizing glass networks. In vitro studies demonstrate that such glasses are biocompatible to tumor and normal cells and that they can promote osteogenic cell proliferation, differentiation, and mineralization. Upon illumination with near-infrared (NIR) light, the bioglass (BG) can efficiently kill bone tumor cells, as demonstrated via in vitro and in vivo experiments. This indicates excellent potential for the integration of multiple functions within the new materials, which will aid in the development and application of novel biomaterials.

## Materials: New material can kill bone tumors and repair bone tissues

A new material, that is not only able to treat bone tumors but can also repair bone tissue, could lead to a more effective treatment for bone cancer. Bone tumor surgery can often leave large defects in bones, which require separate operations involving heat treatment and remineralization to repair. Combining these functions into one operation, however, has proved challenging. Now, Mingying Peng and colleagues from The State Key Laboratory of Luminescent Materials and Devices in China, working with fellow Chinese and American researchers, have developed a new type of bioactive glass that integrates both functions into one material. By illuminating bismuth-doped bioglass with near-infrared light, the researchers have developed a new technique that can kill bone tumor cells and enable photoinduced hyperthermia and bioactivity, reducing the number of treatments required to repair bone tissue.

## Introduction

Bone is a complex type of tissue with the capacity to self-repair^1^. However, regeneration may fail for large bone defects, such as those caused by bone cancer or pathological fractures. For example, common treatments for bone cancer currently involve surgery, chemotherapy, and radiotherapy^2^. These treatments may harm healthy cells in the vicinity of the defect, causing inevitable and permanent defects in the bone tissue. An additional problem can be the presence of residual malignant cells, which may lead to tumor recurrence, significantly affecting treatment prognosis. Therefore, treatments often involve complex secondary surgical operations in which large amounts of bone tissue are removed^3^. This plethora of problems, therefore, calls for multi-functional bioactive materials to aid in tumor treatment and tissue regeneration.

Compared to traditional treatment methods, hyperthermia has been proposed as a minimally invasive way to shorten patient recovery times^4^. It involves the use of functional nanoparticles, laser-induced thermal therapy, or high-intensity ultrasonic treatment^5–8^. In an interesting combination of functions, magnetic bioceramics have been proposed. They combine thermal activity for application in hyperthermia and bioactivity for apatite mineralization, leading to bone tissue repair^9^. For the latter, bioactive glasses have become an attractive alternative, as they provide significantly higher compositional variability and offer the potential for tailored reactivity^10^. Bioceramics and bioglasses (BGs) exhibiting both magnetism and hydroxyapatite (HAp) formation have been investigated for potential application on bone tumors through magnetic hyperthermia^11^. However, various fundamental aspects, such as exposure time, material compatibility, and toxicity of Fe_2_O_3_- or FeO-based approaches, need to be addressed before clinical application can be considered^12^. In addition to magnetic hyperthermia, photothermal (PT) therapy induces thermal apoptosis through heat generated from optical input. Here, the key component is the employed PT conversion agent, which absorbs and converts near-infrared (NIR) light into heat. Various nanoparticle species (NPs) are currently considered as PT agents^13–16^, including, most prominently, metallic NPs but also carbon-based NPs, semiconductor NPs, and organic compounds. In metallic NPs, the thermal effect is generated by localized surface plasmon resonance, which depends on metal or alloy species, particle size, shape, and number density^17–19^. For example, varying the particle shape of Au between rods and shell structures enables a shift in the active spectral range from visible light to NIR; however, this is at the expense of conversion efficiency^20,21^. Carbon-based NPs include carbon nanotubes, carbon nanodots and graphene, which, in addition to having PT functionality, are biocompatible^22–24^. In carbon nanotubes, similar to metallic NPs, light-to-heat conversion is due to the light-induced collective motion of free carriers, that is, excitation of the *π*-plasmon. Problematic to application in hyperthermia, the oxidization process, which is meant to enable dispersion in water, impairs PT efficiency^25,26^.

In the third group of PT materials, semiconductor NPs often provide a significantly higher conversion efficiency than others such as Au NPs. For example, Cu_2−*x*_Se or Cu_9_S_5_ nanocrystals produce PT heating with a conversion efficiency of 22%^27^ and 25.7%^28^, respectively. The main limitation is their well-documented toxicity, especially considering the expectedly high retention time in the body^29^. Furthermore, the spectral mechanism of PT remains unclear; it is either NIR absorption through inter-band electronic transitions or broadband carriers, and this uncertainty makes dedicated tailoring of properties difficult^30^. Organic compounds have also been considered as an alternative with comparably high biocompatibility and biodegradability. Here, problems are primarily related to the poor PT stability^31,32^.

None of these previous strategies, however, can simultaneously integrate PT and bioactivity into one material. Here, we have discovered the first PT effect in bismuth (Bi)-doped glasses. On the basis of this finding, we have developed a new model of Bi-doped bioglass (BG) that simultaneously combines both functions. Generally, once light is absorbed by an optical material, it is very challenging to control radiative and non-radiative processes, which eventually evolve into photoluminescence (PL) and heat, respectively. When heat is accumulated within the matrix, the effect of PT gradually emerges. We find that depolymerizing the matrix glass network can suppress the spontaneous radiation process following NIR excitation; thus it enhances PT conversion efficiency.

In vitro and in vivo experiments were performed to evaluate the biocompatibility, HAp formation, and PT therapeutic ability of Bi-doped BGs. These experiments were designed to investigate the biocompatibility of glasses with both tumor and normal cells and how they promote osteogenic cell proliferation, differentiation, and mineralization on the surface of samples. Under irradiation with NIR light, in vivo experiments with nude mice showed that the materials can efficiently kill bone tumor cells. The model type of Bi-doped BGs can be heated to a desirable temperature range from 38 to 86 °C by either glass components or incident laser power density, and they are bioactive and resorptive. Therefore, they enable remineralization of apatite to facilitate bone regeneration.

## Materials and methods

### Sample preparation

All glass samples were prepared by melting and quenching, and the melting conditions were adapted to the respective glasses. Sample preparation and processing procedures are summarized in the supplementary information, with detailed nominal compositions and melting conditions listed in Table S1. Glass sample codes are shown in column 2 of Table S1. For example, G5AxB indicates (95−*x*) GeO_2_-5 Al_2_O_3_-*x* Bi_2_O_3_ (*x* = 0.05, 0.5, 1.5, 2, 4, 6, 10). All the glass samples were free of bubbles. The sample without Bi appears colorless. Generally, the sample color becomes deeper as the Bi content increases. For example, the germanate glass sample is purple-red when *x* = 0.05, and it becomes reddish brown and even deep reddish brown when *x* = 2 and *x* = 10, respectively. Similar to Bi-doped germanate samples, Bi-doped silicate glass appear reddish brown, while Bi-doped phosphosilicate is light brown. A portion of the glass samples were ground, milled, and sieved using 400 mesh for consequent biological experiments.

### Materials characterization

The PT effect was evaluated by the setup shown in Figure S1, where an 808 nm laser diode (LD) and a ZnSe infrared thermometer (LumaSense IMPAC, Germany), respectively, were used to illuminate samples and monitor the temperature of the sample surface in air in a range from 273 to 1173 K. Here, the 808 nm laser was selected as the irradiation source to avoid water absorption in tissues and improve the tissue-penetration depth^33,34^. The temperature of the samples immersed in simulated body fluid (SBF) solution was measured with a digital thermometer (Kangyou, Nanjing, China). Optical absorption spectra were measured with a Perkin Elmer Lambda 900 UV/Vis/NIR spectrophotometer in a spectral range from 200 to 3200 nm. PL spectra were recorded with a Zolix Omni λ3007 spectrometer equipped with an InGaAs photodetector and a SR830 Stanford lock-in amplifier. The fluorescence lifetimes were measured with a digital phosphor oscilloscope (TDS3012C, Tektronix, America). The excitation spectra were measured with an Edinburgh Instrument FLS 920 (Livingston, WL, UK) equipped with a liquid nitrogen-cooled photomultiplier (Hamamatsu R5509-72, Hamamatsu, Japan).

### Biological experiments

Two representative normal cells, namely, mouse fibroblast (L929)^35^ and murine pre-osteoblast (MC3T3-E1)^36^ cell lines, and two types of tumor cells, namely, rat osteosarcoma-derived (UMR106)^37^ and human osteosarcoma (U2OS)^38^ cells, were purchased from the Type Culture Collection of the Chinese Academy of Sciences. The details are summarized in the supplementary information on cell culture, including in vitro biocompatibility; in vitro mineralization of HAp on BG surfaces in SBF; adhesion, proliferation, differentiation, and mineralization of murine pre-osteoblast cells on Bi-doped BGs; in vitro PT performance of Bi-doped BGs; and in vivo PT therapy experiments on nude mice. All animal studies were approved by the Institutional Animal Care and Use Committee (IACUC) of Guangzhou General Hospital of Guangzhou Military Command. Adult male Balb/c nude mice were purchased from Medical Experimental Animal Center of Guangdong Province.

## Results and discussion

### Discovery of PT effect in Bi-doped germanate glasses

**Fig. 1:**
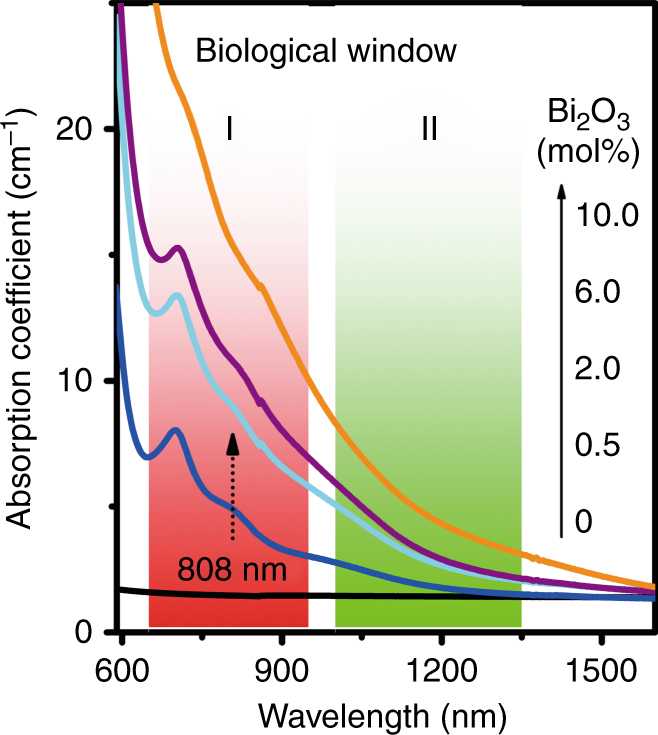
UV to NIR absorption spectra of Bi-doped germanate glass G5AxB as a function of dopant content *x* (labels, mol%). For reference, the first and second biological windows are shaded in red and green, respectively.

Typical materials for PT therapy applications should exhibit NIR absorption peaks within the first and second biological transparent windows (Fig. 1) to combine maximum radiation penetration into the tissue with optimal light absorption of the converter species^17,39,40^. Hence, the overall energy input can be minimized, reducing possible damage to normal tissues during therapy. We occasionally found that, when irradiated by 808 nm LD, all Bi-doped germanate glasses became very hot during investigations of the PL properties. These doped glasses had absorption peaks at ~700 nm, ~800 nm, and ~1000 nm that were clearly absent in the undoped sample G5A0B (Fig. 1). These peaks, according to previous reports, can be assigned to the transition of Bi^+^ from ^3^P_0_ to ^3^P_2_, and the transitions of Bi^0^ from ^4^S_3/2_ to ^2^D_3/2_(2) and ^2^D_3/2_(1), respectively^41,42^. The absorption coefficient at 808 nm increases gradually from ~4.9 cm^−1^ to ~ 15.4 cm^−1^ with an increase in the Bi_2_O_3_ concentration from 0.5 to 10 mol%. Thus, Bi_2_O_3_ addition triggers the desired NIR absorptions, which are primarily located in the first and second biological windows (Fig. 1). This is a prerequisite for PT conversion.

**Fig. 2:**
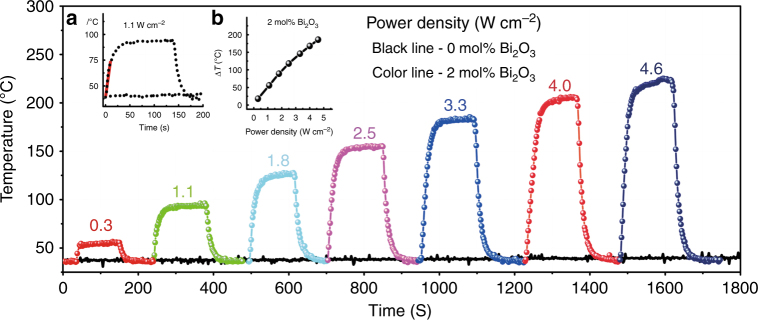
Discovery of PT effect in Bi-doped germanate glasses. Temperature of glass sample G5A2B (colored lines) and blank reference sample G5A0B (black line) as a function of irradiation time and incident power density (label, in W cm^−2^) of CW 808 nm LD. Each curve represents the average of seven on/off cycles. Inset **a** shows a magnification of the data at 1.1 W cm^−2^ irradiation, while inset **b** illustrates the induced temperature increase Δ*T* of glass sample G5A2B as a function of laser power density.

To quantify the PT effect, we set up a device, as illustrated in Figure S1, to detect the surface temperature of Bi-doped germanate glass samples G5AxB (for sample codes, see Table S1). Samples were continuously irradiated until they reached a steady thermal state, followed by cooling to room temperature upon laser shut-off. When samples were irradiated by the 808 nm laser, the temperature of G5A2B increased to 54, 93, 126, 155, 183, 205, and 223 °C as the incident power density increased to 0.3, 1.1, 1.8, 2.5, 3.3, 4, and 4.6 W cm^−2^, respectively. In contrast, under the same conditions of laser irradiation, significant temperature variation did not appear in the blank sample, G5A0B. For example, at a power density of 1.1 W cm^−2^, the temperature of sample G5A2B increases approximately linearly from 37 °C (simulating human body temperature) to 73 °C at a rate of 4.6 °C s^−1^ in the first 10 s (red line, inset (a) of Fig. 2). Subsequently, the temperature reaches a plateau at ~93 °C. In comparison, Au nanoshells, or nanorods, have been reported to provide a typical temperature increase of 15 °C and 22 °C, respectively, after 5 min irradiation under similar illumination conditions (1 W cm^−2^, 800 nm)^27^. In the present case, a steady state was reached after ~90 s; however, this depended on sample geometry and the heat exchange parameters of the surrounding medium. Importantly for potential applications, within the considered range of illumination power density, a linear correlation (*R* = 0.995) was observed between induced temperature increase and power density (inset (b) of Fig. 2).

### Extension of PT effect to Bi-doped silicate glasses

**Fig. 3:**
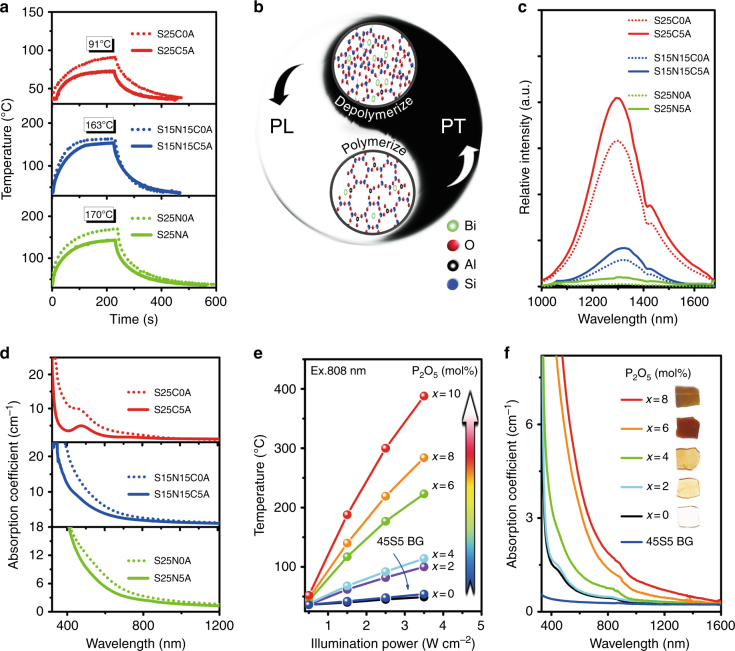
Extension of PT effect to Bi-doped silicate glass, where PT effect could be enhanced by depolymerizing the glass network, and invention of Bi-doped phosphosilicate BGs with pronounced PT effect. **a** Temperature curves of Bi-doped silicate glass samples S25CxA, S15N15CxA, and S25NxA (for sample codes, see Table S1) under 1.5 W cm^−^^2^ irradiation of 808 nm LD; **b** strategy to modulate PT efficiency; **c** emission spectra upon an excitation of 808 nm; **d** absorption spectra of S25CxA, S15N15CxA, and S25NxA; **e** PT effect of Bi-doped phosphosilicate BG samples SxP2B as a function of illumination power and the content x of P_2_O_5_; and **f** absorption spectra of SxP2B and commercial BG 45S5.

Obviously, the germanate glass studied thus far is only a model material, as it does not provide the bioactivity, such as HAp precipitation, that is required for bone repair^43^. Next, we extended the study from germanates to the more technically relevant group of silicates. A series of Bi-doped silicate glasses were made, and the melting conditions and sample codes are compiled in Table S1. Upon laser irradiation (808 nm, 1.5 W cm^−^^2^), steady-state temperatures of ~91 °C, 163 °C, and 170 °C were reached (Fig. 3a). This indicates that the PT effect can be extended to Bi-doped silicate glasses. We also observed that in the corresponding Al_2_O_3_-free samples, this temperature was ~20 K higher (Fig. 3a).

### Strategies to improve PT efficiency

#### Quenching luminescence

**Fig. 4:**
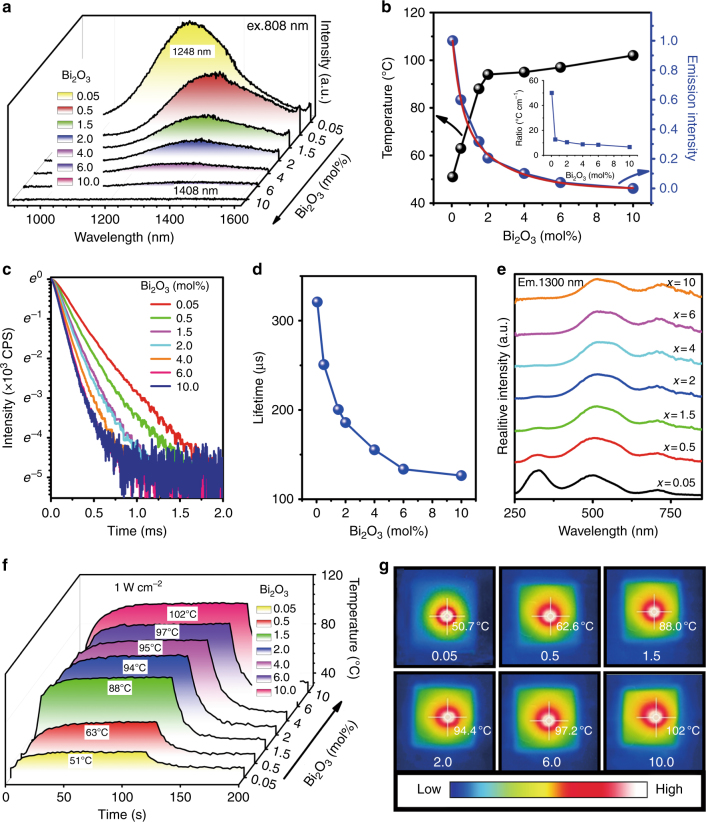
Enhanced PT effect by quenching luminescence in Bi-doped germanate glass. **a** Emission spectra of glass samples G5AxB, where the Bi_2_O_3_ content *x* changes from 0.05 to 10 mol%; **b** temperature (black line) and integrated emission intensity (blue line) of glass samples G5AxB; the red line in **b** demonstrates the double exponential fitting curve with a correlation coefficient of 99.5 %; the inset in **b** demonstrates the ratio of induced temperature increase (Δ*T*) to absorption coefficient at 808 nm (taken from Fig. 1) as a function of Bi_2_O_3_ concentration; **c** decay curves of G5AxB; **d** lifetime of G5AxB with emission at 1300 nm upon 808 nm; **e** excitation spectra (*λ*_em_ = 1300 nm) of G5AxB; **f** temperature curves of G5AxB as a function of irradiation time; power density of 808 nm LD was attenuated to 1 W cm^−^^2^; and **g** thermal images of G5AxB, as the sample temperature became saturated.

As noted in the previous section, the PT effect of Bi-doped germanate glasses arose from the strong absorption bands in the NIR spectral region. As samples were irradiated, active species of Bi within them absorbed these photon energies and then enabled transition to higher excited states. Subsequent relaxation can occur through radiative and non-radiative contributions (Fig. 3b). The latter is associated with energy transfer among Bi ions, multiphonon relaxation, or both, which result in heat generation. Therefore, the thermal effect should be enhanced once PL is suppressed. At high concentrations of Bi_2_O_3_, the Bi centers are sufficiently close to allow inter-ionic energy transfer and, thus, luminescence quenching^44^. In the G5AxB system, this is clearly demonstrated by increasing the concentration of Bi_2_O_3_. The corresponding NIR PL emission spectra from these lower-valent Bi ions are shown in Fig. 4a^42^. As expected, the emission intensity decreases monotonically with increasing concentrations of Bi_2_O_3_, with a stretched-exponential correlation. This is a clear sign of quenching induced by concentration, which is further supported by the dramatic reduction of lifetime from 320.9 μs to 126.5 μs (Fig. 4a–d). At the same time, emission peaks shift from 1248 nm to 1408 nm, and the red-shift is possibly due to enhanced reabsorption. As Figs. 1 and 4a illustrate, the absorption spectra overlap with the emission spectra. The shift might also be influenced by the formation of different emission centers, as illustrated by the excitation spectra (Fig. 4e). Corresponding to luminescence quenching, we expect a parallel increase in the thermal contribution of the relaxation reaction. This is shown in Fig. 4f and g. The observed trends are similar to previous observations of G5A2B (see Fig. 2). For the highest doping concentration (10 mol% of Bi_2_O_3_), the highest temperature of ~102 °C was reached (for a power density of 1 W cm^−2^). As observed from the thermographs, the hottest spot was typically in the center of the irradiated region. With increasing Bi_2_O_3_ concentrations beyond ~0.5 mol%, the maximum temperature did not vary notably, but the heated volume increased (Fig. 4g). This is a logical result of the increasing heat generation. The concrete temperature gradient is a simple result of thermal conductivity and heat exchange at the sample surface. The change in luminescence between the samples does not provide direct information about variations in PT efficiency because the absorption coefficient at 808 nm also increases with Bi concentration. Therefore, the data were normalized to the absorption coefficient at 808 nm, as shown in the inset of Fig. 4b. Clearly, it deviates from a constant line, implying a nonlinear conversion from PL to thermal relaxation as the Bi concentration increases. Although a lower concentration of Bi can lead to a higher normalized heating efficiency, the weak absorption of samples with lower concentrations of Bi cannot fully absorb the incident light, preventing full utilization of the input energy. Thus, illumination only produces the lowest temperature increase for the 0.05 mol% of Bi_2_O_3_ sample. A trade-off must be considered between heating efficiency and absorption coefficient to reach a desirable temperature increase.

Clearly, in Bi-doped germanate glasses, quenching luminescence is an effective strategy to improve the PT efficiency, and it also works well in Bi-doped silicate glasses. For instance, Bi-doped sodium calcium silicate glasses, under irradiation by 808 nm LD with 0.9 W cm^−^^2^, the sample temperature increases from 79 °C to 162 °C as Bi_2_O_3_ content increases from 1 mol% to 4 mol%.

#### Depolymerizing network

According to previous studies, Bi PL strongly depends on glass network integrity, since higher polymerization can efficiently isolate Bi from the surroundings, leading to stronger PL^45^. A backbone network of silicate glass in which Bi resides after doping is constructed by corner-sharing SiO_4_ tetrahedra. The polymerization can be modulated by the addition of species such as Na_2_O, CaO, or Al_2_O_3_^46–48^. Considering the trade-off between PL and PT, here, we investigate the effect of these species on PT. As Fig. 3a depicts, glass samples with no aluminum, but including sodium, exhibit a higher PT effect upon the same illumination than samples with aluminum and/or calcium. Correspondingly, PL becomes much weaker or is quenched by, for example, sample S25N0AS. Therefore, with respect to PT efficiency, a higher content of Na_2_O and the absence of Al_2_O_3_ appear to be most beneficial. To understand this phenomenon, we measured the absorption spectra (Fig. 3d). Samples S25CxAS, which do not contain Na_2_O, show the strongest absorption at ~470 nm, and the absorption at 700 nm originally observed in germanate glass emerges now in the tail of ~470 nm absorption peak. When alumina is added, the absorption coefficients decrease, similar to a previous study on NIR PL^49^. This is partially due to Bi oxidation by an increase in melt basicity. The addition of Al_2_O_3_, as solid state ^27^Al NMR spectra demonstrate, can increase network connectivity; thereby, it can topologically isolate Bi species from each other^45^. This increases the probability of radiative transitions and, in turn, suppresses PT efficiency. Thus, alumina is not suggested for the design of glass with better PT performance, and it is not considered in subsequent experiments. For glass samples free of alumina, we found that the absorption coefficient at 808 nm increases slightly with increasing Na_2_O from 2.09 to 2.43 cm^−1^, for example, for samples S25C0A and S25N0A, respectively. This is a reason that the sodium samples present a better PT property (Fig. 3a). From the microstructural point of view, introduction of sodium can efficiently depolymerize the glass network by forming non-bridged oxygen. This clearly facilitates the processes of PT and, therefore, extinguishes PL, as Fig. 3a–c illustrates.

### Invention of Bi-doped phosphosilicate BGs with pronounced PT effects

As Fig. 3a demonstrates, glass sample S25N0A exhibits the best PT effect. Then, we designed a series of glass samples SxPyB (Table S1) by adding calcium and phosphorus, as these are necessary elements to regenerate bone tissues^43^. We sought samples with functionality in killing residual tumor cells and, consequently, promoting bone repair by remineralization of hexagonal HAp of Ca_10_(PO_4_)_6_(OH)_2_. During glass design, alumina was intentionally omitted due to its counteractive contribution to PT efficiency (as noted above) and potential biological toxicity, and the content of sodium was very close to S25N0A. Calcium and phosphorus were designed to replace SiO_2_ and therefore reduce the content of glass former ions. Therefore, the glass network can be further depolymerized, and the effect of PT can then be enhanced as Fig. 3b demonstrates. The contents of phosphorus and Bi are modulated slightly, as Table S1 illustrates, to achieve the best compromise between PT and bioactivity.

As shown in Fig. 3e, PT heating becomes increasingly efficient with increasing P_2_O_5_, reaching a steady-state temperature of ~188 °C at a power density of 1.5 W cm^−2^ for 10 mol% of P_2_O_5_. This effect can be readily explained by absorption spectra (Fig. 3f), where increasing P_2_O_5_ leads to strongly increasing absorption coefficients, with samples literally becoming black. This occurs because of increasing acidity of melts upon phosphate addition, leading to a reduction of Bi to the lower or even metallic state. Generally, the sample temperature increases rapidly as the power density increases (Fig. 3e). For instance, the temperature of a sample containing 10% P_2_O_5_ can reach 388 °C in air at 3.5 W cm^−2^. Such a significant temperature increase does not appear in commercial BG 45S5 (blue curve in Fig. 3e). The tight dependence of the PT effect on the glass compositions and incident power density reveals that it is very convenient to achieve proper temperatures by simply adjusting these parameters. This implies that less energy is needed for our glasses to reach a temperature sufficient to kill bone tumor cells due to improved PT efficiency after glass design.

### In vitro and in vivo biological experiments with Bi-doped BGs

#### In vitro biocompatibility of Bi-doped BGs to normal and tumor cells

**Fig. 5:**
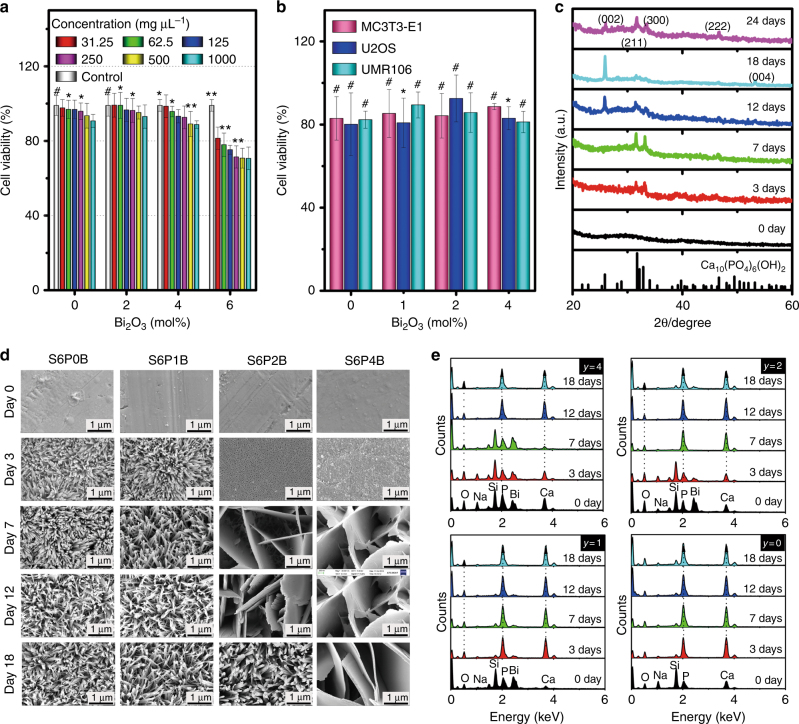
In vitro biocompatibility and mineralization of Bi-doped BG samples S6PyB in SBF solution. **a** Viability of mouse fibroblast cell line L929 after culturing with S6PyB (*y* = 0, 2, 4, 6 mol %) for 24 h; labels in **a** denote concentrations of powder suspensions in mg µL^−1^; **b** viability of normal cell MC3T3-E1, and tumor cells of human osteosarcoma line U2OS and rat osteosarcoma cell lines UMR106 after co-culturing with S6PyB; data points represent the mean values and error bars according to three independent experiments; **c** XRD patterns of sample S6P2B after immersion in SBF for different times as indicated, standard data of JCPDS Card No. 03-0747 of Ca_10_(PO_4_)_6_(OH)_2_ (HAp) is listed as reference at the bottom; **d** morphological evolution of glass samples S6PyB as incubated in SBF for different days as indicated; **e** evolution of element redistribution on sample surfaces of S6PyB as incubated in SBF for different days.

As a first step towards possible biomedical consideration, the potential toxicity of the glasses must be evaluated, especially after Bi doping. Mouse fibroblast cell line (L929), murine calvarial pre-osteoblast (MC3T3-E1) cells, rat osteosarcoma-derived (UMR106) cells, and human osteosarcoma (U2OS) cells were selected as representatives for normal and bone tumor cells for the study on biocompatibility. Generally, the cell viability (%) was >80 % for the four types of cells, L929, MC3T3-E1, UMR106, and U2OS, after culturing with different concentrations of S6PyB glass for 24 h (Fig. 5a,b). The cell viability decreased slightly compared to that of control samples as the suspension concentration of S6PyB glass powders increased from 31.25 mg µL^−1^ to 1000 mg µL^−1^. For glass sample S6P6B, where the Bi_2_O_3_ concentration was 6 mol%, the cell viability was found to decrease from 81 % to 71 % as the power suspension concentration increased to 1000 mg µL^−1^. This indicates that glass S6P6B becomes toxic for L929 cells. Therefore, in the following, the sample with *y* = 6 mol% was not further considered.

#### In vitro mineralization of Bi-doped BG samples S6PxB in SBF solution

To test the bioactivity of the present material, we first examined HAp mineralization during exposure to SBF. Figure 5d depicts field emission scanning electron microscopy (SEM) micrographs of the mineral deposit, which was found on the material surface after immersion in SBF. A rod-like morphology (with a rod diameter of ~50 nm) was observed on the surface of the S6PyB samples (*y* = 0, 1 mol%) after 3 days in SBF. There was no visible difference between these two samples for all exposure times, that is, in the absence of Bi_2_O_3_ or for a Bi_2_O_3_ content of 1 mol%. At higher Bi content, significant differences were observed between the samples. That is, for *y* = 2–4 mol%, the initial porous microstructure (3 days immersion) transforms into an assembly of platelets after 7 days of exposure. The rod-shaped morphology was observed only after 12 days or never for *y* = 4 mol%. Clearly, the larger surface area of the rod-shaped clusters accelerates the corrosion reaction relative to the platelet morphology.

The corrosion reaction occurs for Bi-doped BGs, as such glasses are soaked in SBF solution. Once corrosion starts, the glass surface turns from smooth to porous or rough, which is followed by precipitation and growth of a new phase, that is, HAp Ca_10_(PO_4_)_6_(OH)_2_ (Fig. 5c, d). As soon as the phase of HAp appears and anchors on the sample surface, elements such as P, Ca, and O will diffuse in the vicinity from either SBF solution or glass, promoting further growth of HAp specifically and the mineralization processes generally (Fig. 5c, e). The corrosion reaction, which usually destroys the glass surface and leads to the loss of elements, threatens longevity of the BGs, but it induces bone regeneration and repair.

The results of energy-dispersive X-ray spectroscopy (EDS) analyses are shown in Fig. 5e. After 3 days of immersion, P and Ca are the primary cation species found in the surface deposit, with significant traces of the other glass components still present, especially for the higher content, *y*, of Bi. After 7 days of incubation, the traces of residual glass practically disappear, which indicates the formation of HAp. The addition of Bi_2_O_3_ appears to delay this corrosion and remineralization reaction to a certain extent. The delay enables the precipitation of larger platelets, thereby promoting the incidence of light onto the sample surface through the spaces between these platelets (Fig. 5d). This eventually leads to an improved PT effect compared to the blank sample, where light propagation is blocked by the dense rod assembly covered on the surface (Fig. 5d). Such PT effects can persist even as the samples have been soaked in SBF for 30 days, although it is slightly weakened as the soaking time is prolonged (Figure S2). This should be due to the formation of a HAp layer coated on the sample surface, which may attenuate incident light. The enhanced and persistent PT effect will efficiently and completely extinguish residual bone tumors in the initial state of mineralization. At a later state, platelets of HAp evolve into densely assembled rods on the sample surface, and this strengthens the regenerated bone tissue (see Fig. 5d for the SEM image for 2 mol% Bi_2_O_3_ sample soaked in SBF for 18 days).

#### Adhesion, proliferation, differentiation, and mineralization of murine pre-osteoblast cells on Bi-doped BGs

**Fig. 6:**
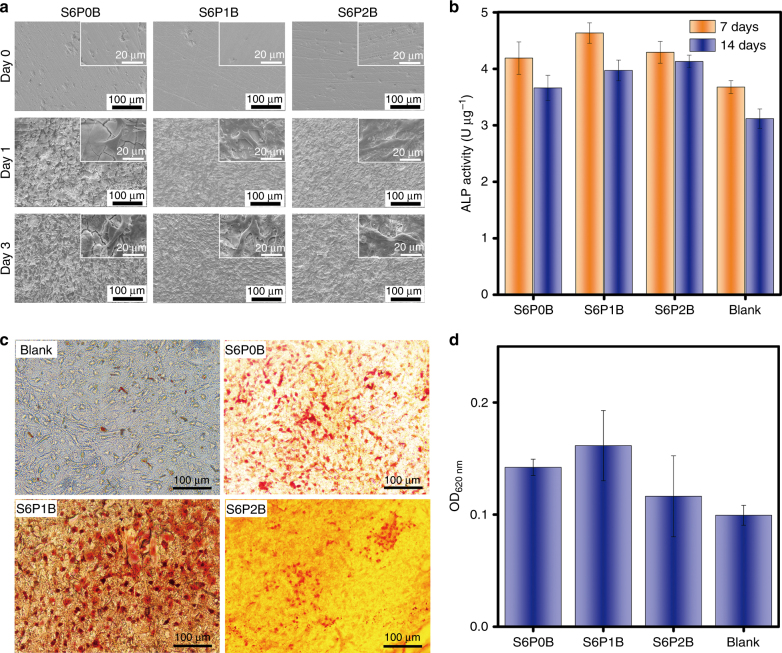
Adhesion, proliferation, differentiation, and mineralization of murine pre-osteoblast cells on Bi-doped BGs. **a** Adhesion and proliferation of MC3T3-E1 cells on the sample surfaces of S6PyB (*y* = 0, 1, 2 mol%) co-cultured for different days; **b** ALP activity of MC3T3-E1 cells cultured with S6PyB for 7 days and 14 days, respectively; **c** in vitro mineralization of osteoblast cells, reflected as calcium deposition on the surfaces of S6PyB, and blank sample co-cultured with MC3T3-E1 cells for 14 days and stained with Alizarin Red S; and **d** OD at 620 nm of S6PyB and blank sample co-cultured with MC3T3-E1 cells for 14 days, stained and dissolved by cetylpyridinium chloride. All data represent the mean values and error bars according to three independent experiments.

In addition to biocompatibility, Bi-doped BGs should enable pre-osteoblast cells to land, populate and mineralize into bone cells, which will eventually regenerate and repair the bone tissues^10^. Therefore, we chose MC3T3-E1 as representative pre-osteoblast cells for this study. After the cells were co-cultured with these glasses in Dulbecco’s minimum essential medium (DMEM) for 1 day, more cracks appeared on the surface of sample S6P0B than on S6P1B or S6P2B. At the same time, the osteoplastic cells started to populate throughout the originally smooth surfaces of these glasses and stick tightly to them (Fig. 6a). At day 3 of co-culture, these cells proliferated quickly and the cell density increased. The surface of S6P0B also became rougher, and gaps between cracks were larger. Cracks started appearing on S6P2B, which is shown in Fig. 5d.

At the early stage of osteogenesis, alkaline phosphatase (ALP) appears in these osteoplastic cells and catalyzes bone cell differentiation^50^. The value of ALP can be observed in the bone cells MC3T3-E1. As Fig. 6b demonstrated, the bone cells exhibited higher ALP values on Bi-doped BGs than the blank or sample S6P0B without Bi on day 7 and day 14. For all samples, the value of ALP on day 14 was lower than that on day 7, perhaps because the cells entered the phases of extracellular matrix maturation or mineralization for bone formation.

In vitro mineralization of osteoblast cells was indicated by calcium deposition (or nodules formed by osteogenic cells) on the surfaces of S6PyB and blank samples co-cultured with MC3T3-E1 cells for 14 days and stained with Alizarin Red S (Fig. 6c). Bi-doped BG, especially S6P1B, exhibited the highest calcium deposition or the most nodules on the surface of all samples (Fig. 6c, d). As Fig. 6d demonstrated, it was 1.62 times higher than the blank control, where no BG was applied.

These in vitro experiments demonstrate that the Bi-doped BGs exhibit excellent biocompatibility with pre-osteoblast cells, and they can facilitate cell proliferation and osteogenic differentiation and mineralization.

#### In vitro PT effect of Bi-doped BGs

**Fig. 7:**
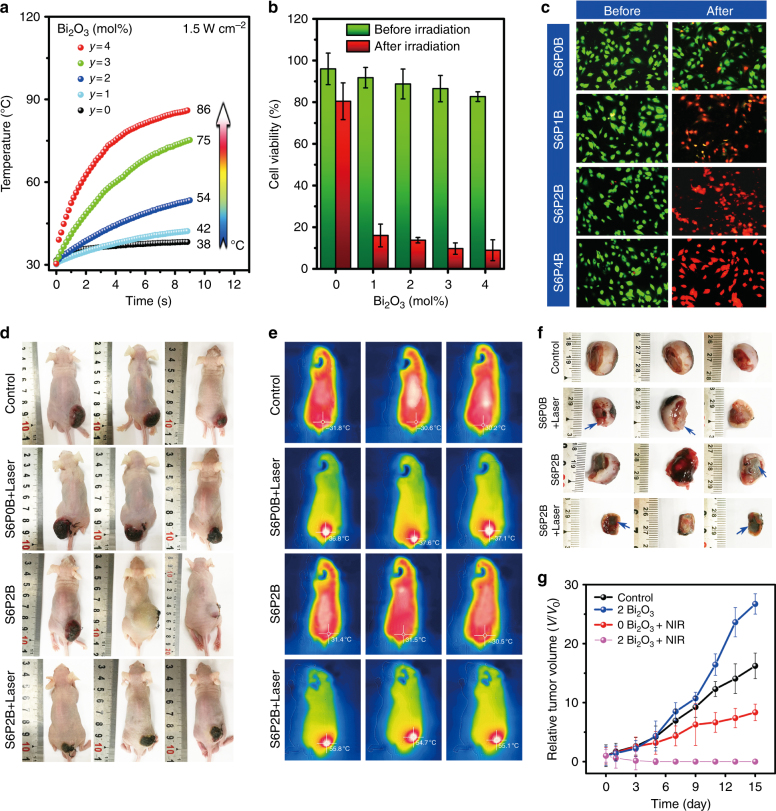
In vitro and in vivo PT tumor therapy of Bi-doped BGs. **a** Temperature curves of BG samples S6PyB immersed in SBF solution for different irradiation times at a power density of 1.5 W cm^−2^; **b** cell viability of S6PyB before and after 808 nm laser irradiation at 1.5 W cm^−2^ for 5 min; data represent the mean values and error bars according to three independent experiments; **c** fluorescence microscopic images of human osteosarcoma line U2OS incubated with S6PyB before and after 808 nm laser irradiation at 1.5 W cm^−2^ for 5 min; live cells are green, while dead cells are red due to the fluorescence of calcein and PI-DNA upon the illumination of 490 nm; **d** images of mice in “control”, “S6P0B + laser”, “S6P2B” and “S6P2B + laser” groups at day 15, **e** thermal images of mice in the four groups at day 0; **f** tumor tissues of mice in the four groups at day 15 where the blue arrows indicate the location of the glass; and **g** tumor volume evolution of the four groups with times.

We tested hyperthermia in vitro, using S6PyB (*y* = 0, 1, 2, 4 mol%) as the PT material, in turn, with SBF, methylthiazolyldiphenyl tetrazolium bromide (MTT) assay, and calcein-AM/PI staining for live/dead staining. As Fig. 7a demonstrated, the temperature of S6PyB increased from 42 °C to 86 °C when the concentration of Bi_2_O_3_ in the glass increased from 1 to 4 mol%. In the undoped control, there is practically no temperature effect. The decrease in heating kinetics is caused by the improved heat transfer at the sample surface because of SBF immersion. At this point, it seems very feasible to generate the desired heating effect necessary for hyperthermia applications using the present BG. Human osteosarcoma line U2OS cells were selected as the most typical bone tumor cells for PT therapy. Standard MTT assays were carried out to determine the relative viabilities after 24 h of incubation (Fig. 7b and Figure S3). All samples exhibited non-cytotoxicity without laser irradiation. After irradiation with an 808 nm laser, more than 80 % of the human osteosarcoma line U2OS cells were killed by S6PyB (*y* = 1, 2, 3, 4 mol%) samples, whereas the cell viability in the undoped control was only decreased by 15 %. This effect was further investigated and confirmed by fluorescence microscopy. Clearly, cell mortality gradually increased with the concentration of Bi_2_O_3_. The tumor cells incubated with the S6P1B glass sample could reactivate, but 2–4 mol% of Bi_2_O_3_ led to destructive death (Fig. 7c). This is clear evidence for the potential of hyperthermia combined with a BG material.

#### In vivo PT therapy experiments on nude mice

To further demonstrate possible in vivo applications, four parallel in vivo experiments were performed with nude mice, including “control”, “S6P0B + laser”, “S6P2B”, and “S6P2B + laser” groups. For the “control” group, no glass or laser irradiation was applied. As the rat osteosarcoma derived from UMR106 cells grew to ~10 mm, glass samples were implanted into the lower tumors of mice (Fig. 7d). Hematoxylin and eosin (H&E) stain analyses demonstrated that implanting Bi-doped or undoped glass samples for 15 days did not lead to damage or inflammation of mice organs, such as heart, liver, spleen, lung, or kidney (Figure S4). The temperature of the tumor site lies between 30 and 32 °C, and the implanted BG did not produce a noticeable difference between groups of “control” and “S6P2B,” if individual differences between mice were neglected (Fig. 7e). Regarding laser illumination of the tumors, the temperature of the “S6P0B + laser” group increased by 6–7 °C while “S6P2B + laser” group soon reached ~55 °C (Fig. 7e). The laser illumination persisted for only 10 min at a power density of 1.5 W cm^−2^ before it was switched off. There was an obvious difference between these groups. Day 15 tumors in the “control”, “S6P0B + laser”, and “S6P2B” groups became larger (Fig. 7d, f, g), and tumor cells were alive based on the H&E stain analyses (Figure S4). However, tumors in the “S6P2B + laser” group started disappearing on day 1, and they vanished at day 3. At day 15, all tumors were destroyed (Figure S4), and the only BGs observed at original tumor sites and normal muscular tissues had already started growing on the surfaces, wrapping around them. This demonstrates in vivo biocompatibility and tumor therapy potential for such glasses.

## Conclusions

In summary, after the discovery of the PT effect in Bi-doped glasses, we determined that such an effect can be effectively controlled by quenching luminescence using the active Bi species or depolymerizing the glass network. Consequently, we have developed a new type of Bi-doped bioactive phosphosilicate glass, which, as in vitro experiments demonstrate, combines high PT conversion efficiency, biocompatibility, and HAp remineralization to reduce the number of treatment cycles required to treat large bone defects. The glasses also allow the population, differentiation and mineralization of osteogenic cells on their surfaces, demonstrating proof of concept to repair bone tissues. Both in vitro and in vivo experiments indicate that these glasses can efficiently destroy bone tumor cells upon illumination with NIR light and also promote regrowth of the muscle tissue around them. H&E stain analysis demonstrate that implanting these glasses did not permanently damage mice organs. Thus, Bi-doped BGs assist with toxic chemo-therapies and radiation-therapies, inducing the destruction of residual cancer cells, and they also enable the application of emerging treatment techniques, such as interventional treatment. This indicates an excellent potential for the development and application of multi-functional biomaterials.

## Electronic supplementary material

Supplementary Information(PDF 656 kb)

Supplementary Figure(TIF 3907 kb)

TOC

## Electronic supplementary material

**Supplementary information** accompanies for this paper at 10.1038/s41377-018-0007-z.
