# Correction: Multi-functional bismuth-doped bioglasses: combining bioactivity and photothermal response for bone tumor treatment and tissue repair

**DOI:** 10.1038/s41377-019-0165-7

**Published:** 2019-06-12

**Authors:** Liping Wang, Nicholas J. Long, Lihua Li, Yao Lu, Mei Li, Jiangkun Cao, Yu Zhang, Qinyuan Zhang, Shanhui Xu, Zhongmin Yang, Chuanbin Mao, Mingying Peng

**Affiliations:** 1 The State Key Laboratory of Luminescent Materials and Devices, Guangdong Engineering Technology Research and Development Center of Special Optical Fiber Materials and Devices, Guangdong Provincial Key Laboratory of Fiber Laser Materials and Applied Techniques, School of Materials Science and Engineering, South China University of Technology, 510641 Guangzhou, China; 2 Department of Chemistry, Imperial College London, South Kensington, London, SW7 2AZ UK; 3 Guangdong Key Lab of Orthopedic Technology and Implant Materials, Department of Orthopedics, Guangzhou General Hospital of Guangzhou Military Command, 111 Liuhua Road, 510010 Guangzhou, China; 4 Department of Chemistry and Biochemistry Stephenson Life Sciences Research Center, University of Oklahoma, Norman, OK 73072 USA

**Keywords:** Biomaterials, Biophotonics

**Correction to: Light Science & Applications**

10.1038/s41377-018-0007-z published online 18 May 2018

In this originally published article, we have noticed several mistakes. They should be corrected as follows:

1. On page 1, the second affiliation (No. 5) of the author “Chuanbin Mao” should be deleted as he does not belong to that affiliation. Namely, he should be only listed with (Department of Chemistry and Biochemistry Stephenson Life Sciences Research Center, University of Oklahoma, Norman, OK 73072, USA).
2. On page 3, in the section of “Discovery of PT effect in Bi-doped germanate glasses”, the absorption wavelength in Fig. 1 starts from 600 nm in the visible range. Therefore, “UV” should be “Visible” in the caption of Fig. 1.
3. On page 8, in the section of “In vitro mineralization of Bi-doped BG samples S6PxB in SBF solution”, we put a wrong SEM image in Fig. 5d for glass samples S6P4B which was incubated in SBF for seven days. The whole Figure has been updated as “Fig. 5 correction”.
4. On page 12, in the section of “Author details”, the affiliation (No. 5) should be deleted as the author “Chuanbin Mao” does not belong to that.

**Fig. 5:**
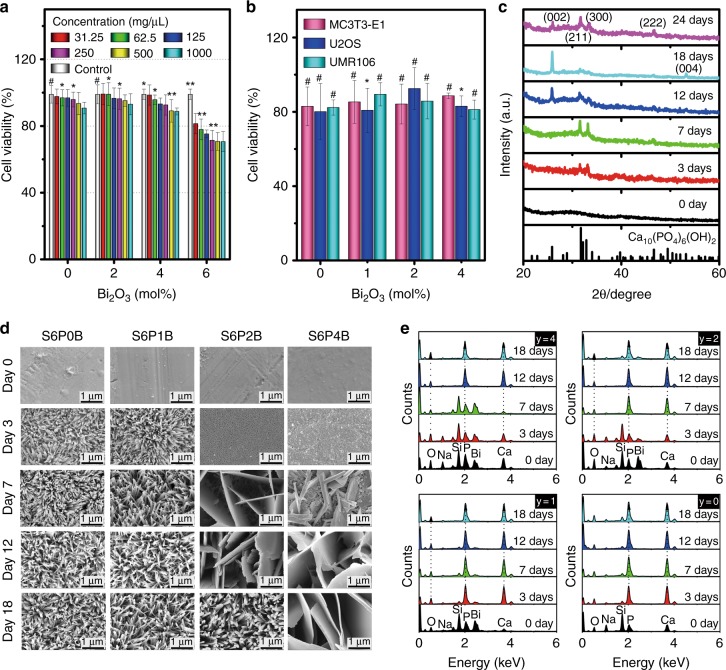
Correction: In vitro biocompatibility and mineralization of Bi-doped BG samples S6PyB in SBF solution. **a** Viability of mouse fibroblast cell line L929 after culturing with S6PyB (y = 0, 2, 4 and 6 mol%) for 24 h; labels in a denote concentrations of powder suspensions in mg/μL. **b** Viability of normal cell MC3T3-E1 and tumor cells of human osteosarcoma line U2OS, and rat osteosarcoma cell lines UMR106 after co-culturing with S6PyB; data points represent the mean values and error bars according to three independent experiments. **c** XRD patterns of sample S6P2B after immersion in SBF for different times as indicated, standard data of JCPDS Card No. 03-0747 of Ca_10_(PO_4_)_6_(OH)_2_ (HAp) is listed as reference at the bottom. **d** Morphological evolution of glass samples S6PyB as incubated in SBF for different days as indicated. **e** Evolution of element redistribution on sample surfaces of S6PyB as incubated in SBF for different days

We really apologize for these mistakes.

