# Supplementary material for: Multi-functional bismuth-doped bioglasses: combining bioactivity and photothermal response for bone tumor treatment and tissue repair
Source: Light Sci Appl. 2018 May 18;7:1. doi: 10.1038/s41377-018-0007-z (PMC6106990; doi:10.1038/s41377-018-0007-z)
Supplement: Supplementary file 1 — Supplementary Information(PDF 656 kb) [file 41377_2018_7_MOESM1_ESM.pdf]

Supplementary Information

Multi-functional bismuth-doped bioglasses: combining bioactivity and photothermal response for bone tumor treatment and tissue repair

Liping Wang[1], Nicholas J. Long[2], Lihua Li[1], Yao Lu[3], Mei Li[3], Jiangkun Cao[1], Yu Zhang[3], Qinyuan Zhang[1], Shanhui Xu[1], Zhongmin Yang[1], Chuanbin Mao[4], Mingying Peng[1,]*

[1]The State Key Laboratory of Luminescent Materials and Devices, Guangdong Engineering Technology Research and Development Center of Special Optical Fiber Materials and Devices, Guangdong Provincial Key Laboratory of Fiber Laser Materials and Applied Techniques, School of Materials Science and Engineering, South China University of Technology, Guangzhou 510641, China.

[2]Department of Chemistry, Imperial College London, South Kensington, London, SW7 2AZ, UK.

[3]Guangdong Key Lab of Orthopedic Technology and Implant Materials, Department of Orthopedics, Guangzhou General Hospital of Guangzhou Military Command, 111 Liuhua Road, Guangzhou 510010, China

[4]Department of Chemistry and Biochemistry Stephenson Life Sciences Research center University of Oklahoma, Norman, OK 73072, USA

E-mail: pengmingying@scut.edu.cn

This PDF includes:

Supplementary Note S1-S7

Supplementary Figure S1-S4

Supplementary Table S1

SUPPLEMNTARY NOTES

MATERIALS AND METHODS

S1 Sample preparation

Glass samples of Bismuth (Bi)-doped germanate, silicate and phosphosilicate were prepared by a technique of melting and quenching. As for raw materials, powders of GeO_2 (99.99%), SiO_2 (99.99%), Bi_2O_3 (99.99%), P_2O_5 (99.8%) and analytical grade Na_2CO_3, $CaCO_3$, $Al(OH)_3$ were employed. Batches of 30 g were prepared for each composition by homogeneously mixing in an agate mortar. These batches were subsequently melted in corundum crucibles in air, and then cast quickly onto a stainless steel plate and pressed immediately with another steel plate to increase cooling rate to prevent possible crystallizations. The glass samples were cut and polished into 10 mm×10 mm×1 mm for consequent measurements. Detailed nominal compositions and melting conditions are summarized in Table S1. Glass samples are coded within column 2 as presented in Table S1. For instance, G5AxB means (95-x) GeO_2-5 Al_2O_3-x Bi_2O_3 (x = 0.05, 0.5, 1.5, 2, 4, 6, 10 mol%). All the glass samples are bubble-free. The sample without Bi appears colorless. Generally, the sample color becomes deeper as the Bi content increases. For instance, the germanate glass sample is purple-red as x= 0.05, and it becomes reddish-brown and even deep reddish-brown as x = 2 and x = 10, respectively. Similarly to Bi-doped germanate samples, Bi-doped silicate glass shows as reddish-brown while Bi-doped phosphosilicate is light brown. A portion of 5 g of glass samples were manually ground into a powder in an agate mortar. A vertical planetary ball mill (XQM systems, Changsha, China) was then used for further milling, and it employed stainless steel containers and balls of hardened steel with a diameter of 70 mm. The glass powders were milled at a rotation frequency of 350 min^{-1} for 12 h in dry state. From the resulting powders, the fraction with diameter less than 400 mesh was selected through sieving for consequent biological experiments.

S2 Cell Culture

Two typical representatives of normal cells such as mouse fibroplast cell line (L929) and murine pre-osteoblast cells (MC3T3-E1), and two types of tumor cells for instance rat osteosarcoma-derived cells (UMR106) and human osteosarcoma line cells (U2OS) were purchased from the Type Culture Collection of the Chinese Academy of Sciences. They were dispersed first in a balanced salt solution containing 0.25 % trypsin and 0.04% EDTA, and separated after centrifugation, and eventually incubated at 37 °C in an atmosphere of 5% CO_2 and

95% air within Dulbecco's minimum essential medium (DMEM, Gibco, USA) into which 10 % fetal bovine serum (FBS, Gibco, USA), 100 U/mL penicillin and 100 mg/mL streptomycin were added.

S3 *In vitro* biocompatibility of Bi-doped BGs

Bi-doped phosphosilicate glasses were first sterilized at 120 ^{o}C for 20 min, and then put into 24-well plates. Each separated well of the plates was filled by 500 μL DMEM containing L929, MC3T3-E1, U2OS or UMR106 cells in a density of 1×10^4 cells/cm^3, and they were co-cultured at 37 °C in 5% CO_2/95% air for 24 h. 40 μL Phosphate buffer saline with 5 mg/mL methylthiazolyldiphenyl tetrazolium bromide (MTT) was added afterwards into each well, and the sample plates were incubated once again for 4 h at 37 ^{o}C. Succinate dehydrogenase produced by these live cells reacted with MTT and led to the formation of blueish violet formazan within cells. The precipitated formazan was dissolved with 400 μL of dimethyl sulfoxide (DMSO) for consequent measurements on optical density at 490 nm by a microplate reader (Thermo, Multiskan GO). The values were averaged from three independent experiments.

S4 *In vitro* mineralization of hydroxyapatite on the surfaces of Bi-doped BGs in simulated body fluid (SBF)

In vitro mineralization processes of hydroxyapatite were simulated in SBF solution on the surfaces of Bi-doped BGs. For this, the polished glass samples were further cut into the size of 5 mm× 5 mm×1 mm. Six blank samples of every glass composition were soaked separately in 10 ml of SBF (pH 7.4) for 0, 3, 7, 12, 18 and 24 days at 37 ^{o}C and 30% relative humidity in temperature- and humidity-controlled chambers (K-Sun Technology, Guangdong, China). The SBF solution was renewed every other day. After the specified immersion periods, the immersed glass samples were removed from the SBF solution, gently rinsed with distilled water, and dried at room temperature. Morphology and composition of the generated crystals on the surface were characterized via a field emission scanning electron microscopy (Merlin-SEM, Zeiss, Germany) equipped by an energy dispersive X-ray spectroscopy (EDS, Oxford, England). The crystalline phases were identified by X-ray diffraction (X'Pert Pro, Panalytical, Netherlands).

S5 Adhesion, proliferation, differentiation and mineralization of murine pre-osteoblast cells on Bi-doped BGs

Murine pre-osteoblast (MC3T3-E1) cells were chosen as model osteoblast cells to study the adhesion, proliferation, differentiation and mineralization of the cells on Bi-doped BGs. Similarly

to the experiments on *in vitro* biocompatibility, the cells of MC3T3-E1 were cultured in DMEM for 0, 1 and 3 days, respectively, with glass samples S6PyB where y = 0, 1, 2 mol% (Table S1). Afterwards, DMEM was removed from the culture wells, and the wells were gently rinsed three times with phosphate buffer saline (PBS) to remove residual DMEM. The wells were refilled in consequence with 500 μL of 2.5% glutaraldehyde solution to fix the cells on the sample surfaces. The samples were washed 3 times with PBS, and dehydrated in a series of solutions with the increase of ethanol content from 30 %, to 50, 70, 80, 90, 95 and 100 %. They were eventually coated with gold for SEM images (EVO18, Zeiss, Germany) to monitor the morphological change and the cell adhesion on sample surfaces.

Glass samples S6PyB (y=0, 1, 2 mol %) were put in the 24-well plates which were filled by 500 μL DMEM with a density of 1×10^4 MC3T3-E1 cells per cm^3. Osteogenic differentiation of MC3T3-E1 cells was induced by addition of 1 % osteogenesis revulsant into DMEM. The osteogenesis revulsant was prepared with 100 mmol/L dexamethasone, 0.05 mmol/L ascorbic acid and 10 mmol/L Na-β-glycerophosphate. As a reference, a control group was prepared in parallel without glass samples. 500 μL triton X-100 was added into each well after removal of DMEM and reacted overnight with MC3T3-E1 cells to release alkaline phosphatase (ALP). 50 μL p-Nitrophenyl phosphate was subsequently added to the solution and reacted with ALP and therefore produced p-nitrophenol. For Day 7 and Day 14 samples, ALP activities were determined with a colorimetric assay[1, 2] by monitoring the optical density at 405 nm. Each experiment was repeated three times and ALP activity was averaged accordingly.

In vitro mineralization of osteoblast cells was evaluated by a technique of Alizarin Red S staining[3]. After the glass samples S6PyB (y=0, 1, 2 mol%) had been co-cultured with MC3T3-E1 cells for 14 days, the samples were washed three times with PBS, and the cells on them were fixed with 75 % ethanol solution for 1 h, and then stained with 40 mmol/L Alizarin Red S (Sigma, pH 4.2) for 30 min at room temperature. After washing to colorless with deionized water, the samples were examined with a microscope objective (Nikon, 50i, Japan) and inverted phase contrast microscope (Olympus, BX51, Japan), respectively, to visualize the distribution of calcium deposits on sample surfaces. The calcium deposits were then dissolved by 10% cetylpyridinium chloride in 10 mmol/L sodium phosphate (pH = 7.0) and the optical density was measured at 620 nm for the quantitative analysis.

S6 *In vitro* PT performance of Bi-doped BGs

Bi-doped phosphosilicate glass samples S6PyB (y=0, 1, 2, 4 mol% in Table S1) were loaded into the 24-well plates and then filled with 400 μL DMEM with a density of 1×10^4 cells/cm^3 for

mouse fibroblast cell line (L929), murine pre-osteoblast cells (MC3T3-E1), and human osteosarcoma line cells (U2OS), respectively. After incubation for 24 h, the samples were treated with and without irradiation of 808 nm laser with a power density of 1.5 W/cm^2 for 5 min. *In vitro* PT performance of Bi-doped BGs was evaluated by a similar technique of MTT[4] to previous *in vitro* biocompatibility. 300 μL PBS with 1 μmol/L calcein acetoxymethyl ester (Calcein-AM) and 2 μmol/L propidium iodide (PI) were added into each well. Live cells in the plates reacted with Calcein-AM at 37 ^{o}C for 15 min, and Calcein was, thus, produced. The dead cells reacted with PI, and PI-DNA was therefore formed. Upon the illumination of 490 nm, Calcein and PI-DNA exhibited emissions at 515 nm and 617 nm, respectively, being clearly distinguished with a fluorescence microscope (Olympus, BX51, Japan).

S7 *In vivo* PT therapy experiments on nude mice

All animal studies were approved by Institutional Animal Care and Use Committee (IACUC) of Guangzhou General Hospital of Guangzhou Military Command. Adult male Balb/c nude mice were purchased from Medical Experimental Animal Center of Guangdong Province. 200 μL DMEM with a density of 5.0×10^6 tumor cells per cm^3 of rat osteosarcoma derived UMR106 cells were injected into the back of nude mice. When the tumor grew to ~10 mm in diameter, the mice were separated into four groups randomly: (i) "control" group where no BGs were inserted, (ii) "S6P0B + laser" group where BGs without Bi were applied with laser irradiation, (iii) "S6P2B" group where BG samples doped with 2% Bi_2O_3 were employed but without laser irradiation, and (iv) "S6P2B + laser" group where BG samples doped with 2% Bi_2O_3 were used with laser irradiation for 10 min. The skins of nude mice were slightly incised along the edge of the tumor and the incision was ~5 mm long without breaking tumor tissues. Pieces of glass samples in 5 mm× 5 mm×1 mm were inserted gently into the bottom of tumor tissues, and the cuts were stitched up afterwards. The stretched cuts could be clearly seen on the back of the mouse in "S6P2B" group because of the tumor growth (Figure 7d). The laser power density of 808 nm LD was attenuated to ≈1.5 W/cm^2 and it was incident on the top of the tumor directly against the glass surface. Tumor temperatures were monitored in real-time by an infrared camera to prevent higher temperature increase during 10 min irradiation. Tumor sizes were measured every other day over 15 days, and the tumor volumes were calculated according to ref. 5. After 15 days, the mice were anesthetized by anhydrous alcohol for euthanasia, and dissected subsequently. The organs were collected and fixed in 4% paraformaldehyde overnight at 4 °C. To examine the impact of implanted BGs on mice organs and tissues, hematoxylin and eosin (HE) staining[6] was performed according to the standard protocols. All images were investigated by microscopy (Olympus, BX51, Japan).

REFERENCES

1 Iqbal J. An enzyme immobilized microassay in capillary electrophoresis for characterization and inhibition studies of alkaline phosphatases. *Anal Biochem* 2011; **414**: 226-231.

2 Thompson RQ, Barone GC, Halsall HB, Heineman WR. *Anal Biochem* 1991; **192**: 90-95.

3 Paul H, Reginato AJ, Schumacher HR. Alizarin red s staining as a screening test to detect calcium compounds in synovial fluid. *Arthritis Rheumatol* 1983; **26**: 191-200.

4 Sgouras D, Duncan R. Methods for the evaluation of biocompatibility of soluble synthetic polymers which have potential for biomedical use: 1-use of the tetrazolium-based colorimetric assay (MTT) as a preliminary screen for evaluation of *in vitro*, cytotoxicity. *J Mater Sci-Mater M* 1990; **1**: 61-68.

5 Osborne CK, Hobbs K, Clark GM. Effect of estrogens and antiestrogens on growth of human breast cancer cells in athymic nude mice. *Cancer Res* 1985； **45**: 584-598.

6 Fischer AH, Jacobson KA, Rose J, Zeller R. Hematoxylin and eosin staining of tissue and cell sections. *Cold Spring Harb Protoc* 2008; **5**: 1-2.

SUPPLEMNTARY FIGURES

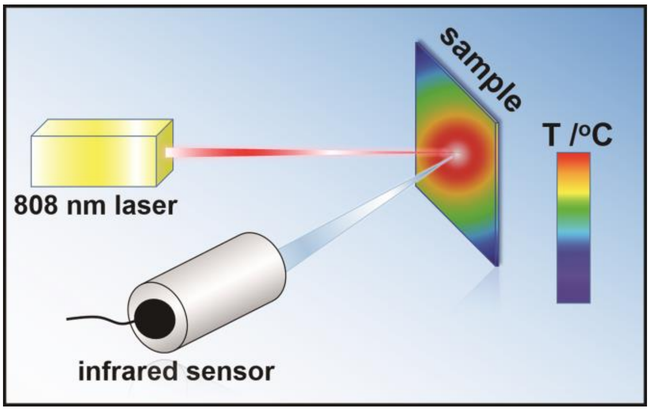

Figure S1. Scheme for quantification of the PT effect.

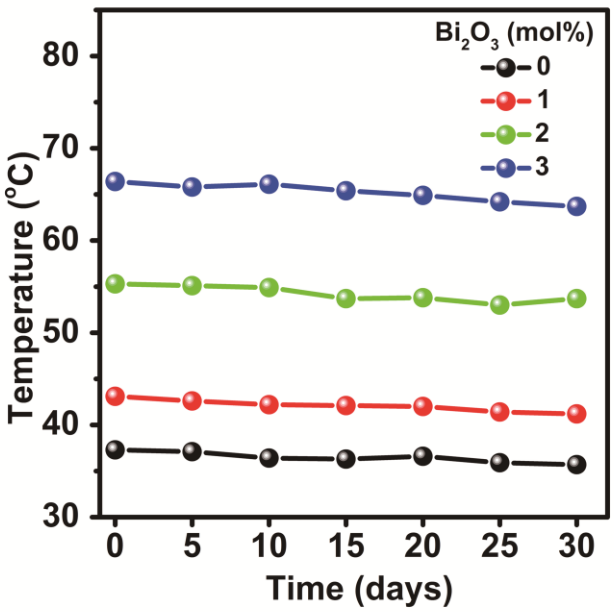

Figure S2. Long run PT effect of Bi-doped BGs S6PyB (y=0, 1, 2, 3 mol%) immersed in SBF solution for different days. The temperature was measured under irradiation of 808 nm LD for 10 min.

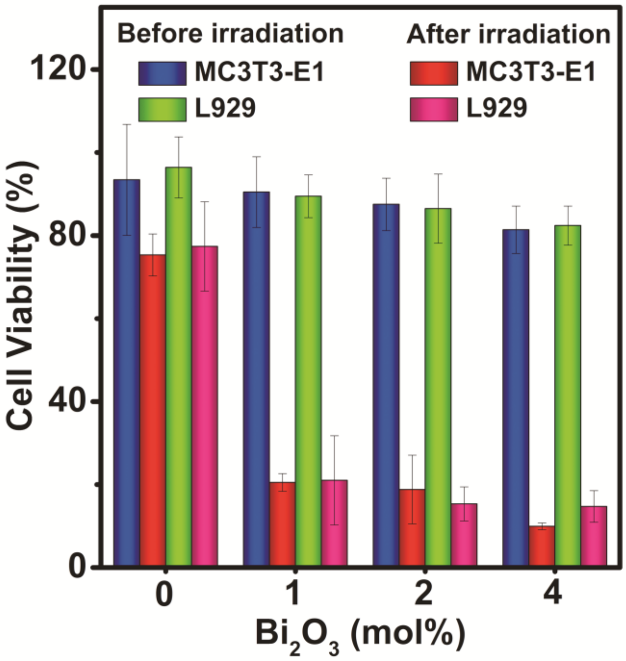

Figure S3. Viability of normal cells MC3T3-E1 and L929 of glass samples S6PyB (y=0, 1, 2, 4 mol%) before and after laser irradiation at 1.5 W/cm^2 for 5 min. The mean values and the variations derive from three independent experiments.

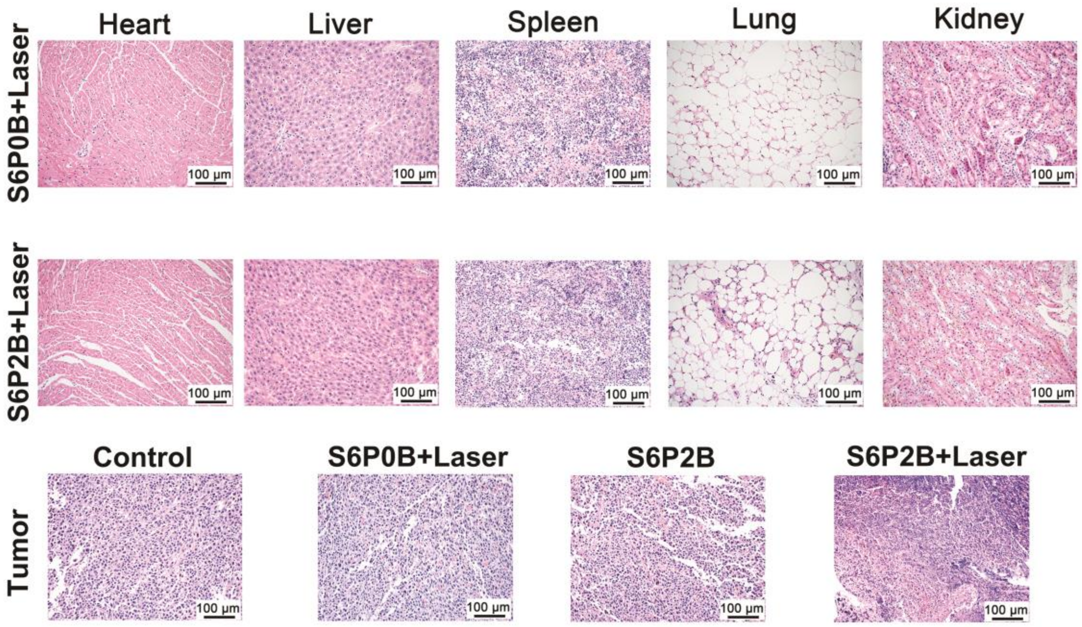

Figure S4. Micrographs of H&E stained organ and tumor slices from samples of different groups as indicated. Organ damage only appeared for tumor slice from “S6P2B + laser” group.

SUPPLEMNTARY TABLES

Table S1. Nominal compositions and melting conditions of considered glass matrices

Glass system	Sample code	Nominal composition (mol %)	Melting temperature (°C)	Melting time (min.)
Germanate	G5AxB	(95-x) GeO_2-5 Al_2O_3-x Bi_2O_3 (x = 0.05, 0.5, 1.5, 2, 4, 6, 10)	1540	30
Silicate	S25CxA	(73-x) SiO_2-2 Bi_2O_3-25 CaO-x Al_2O_3 (x = 0, 5)	1450	60
	S15N15CxA	(68-x) SiO_2-2 Bi_2O_3-15 Na_2O-15 CaO-x Al_2O_3 (x = 0, 5)	1450	60
	S25NxA	(73-x) SiO_2-2 Bi_2O_3-25 Na_2O-x Al_2O_3 (x = 0, 5)	1450	60
Phospho-silicate	SxPyB	(49-x-y) SiO_2-24 Na_2O-27 CaO-x P_2O_5-y Bi_2O_3 (x = 0, 2, 4, 6, 8, 10 , y = 0, 1, 2, 4, 6)	1450	30
