# Supplementary figures and images for: Multi-functional bismuth-doped bioglasses: combining bioactivity and photothermal response for bone tumor treatment and tissue repair
Source: Light Sci Appl. 2018 May 18;7:1. doi: 10.1038/s41377-018-0007-z (PMC6106990; doi:10.1038/s41377-018-0007-z)

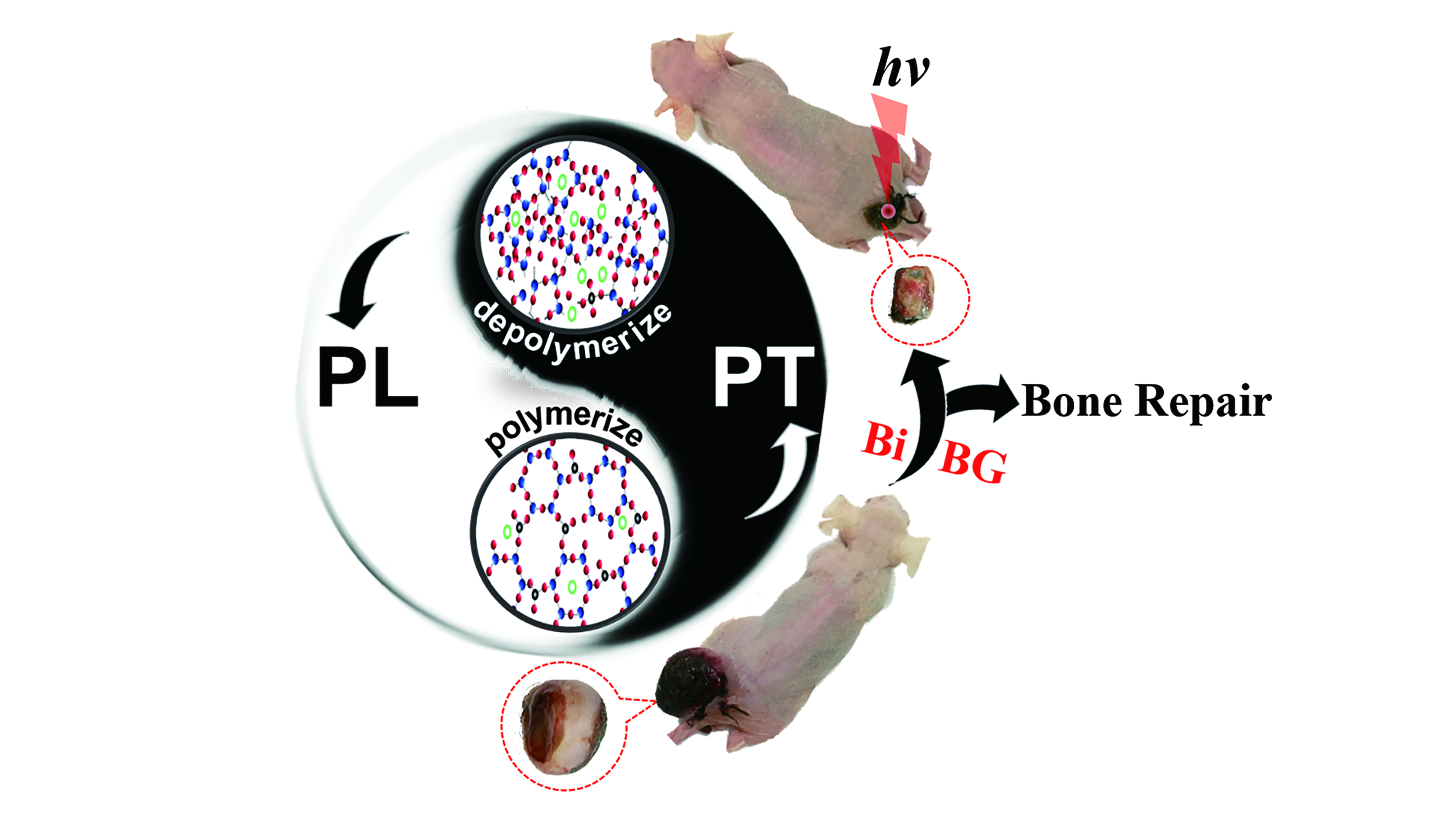

Supplement: Supplementary file 2 — Supplementary Figure(TIF 3907 kb) [file 41377_2018_7_MOESM2_ESM.tif]
