# Supplementary material for: Multi-functional bismuth-doped bioglasses: combining bioactivity and photothermal response for bone tumor treatment and tissue repair
Source: Light Sci Appl. 2018 May 18;7:1. doi: 10.1038/s41377-018-0007-z (PMC6106990; doi:10.1038/s41377-018-0007-z)
Supplement: Supplementary file 3 — TOC [file 41377_2018_7_MOESM3_ESM.doc]

TOC


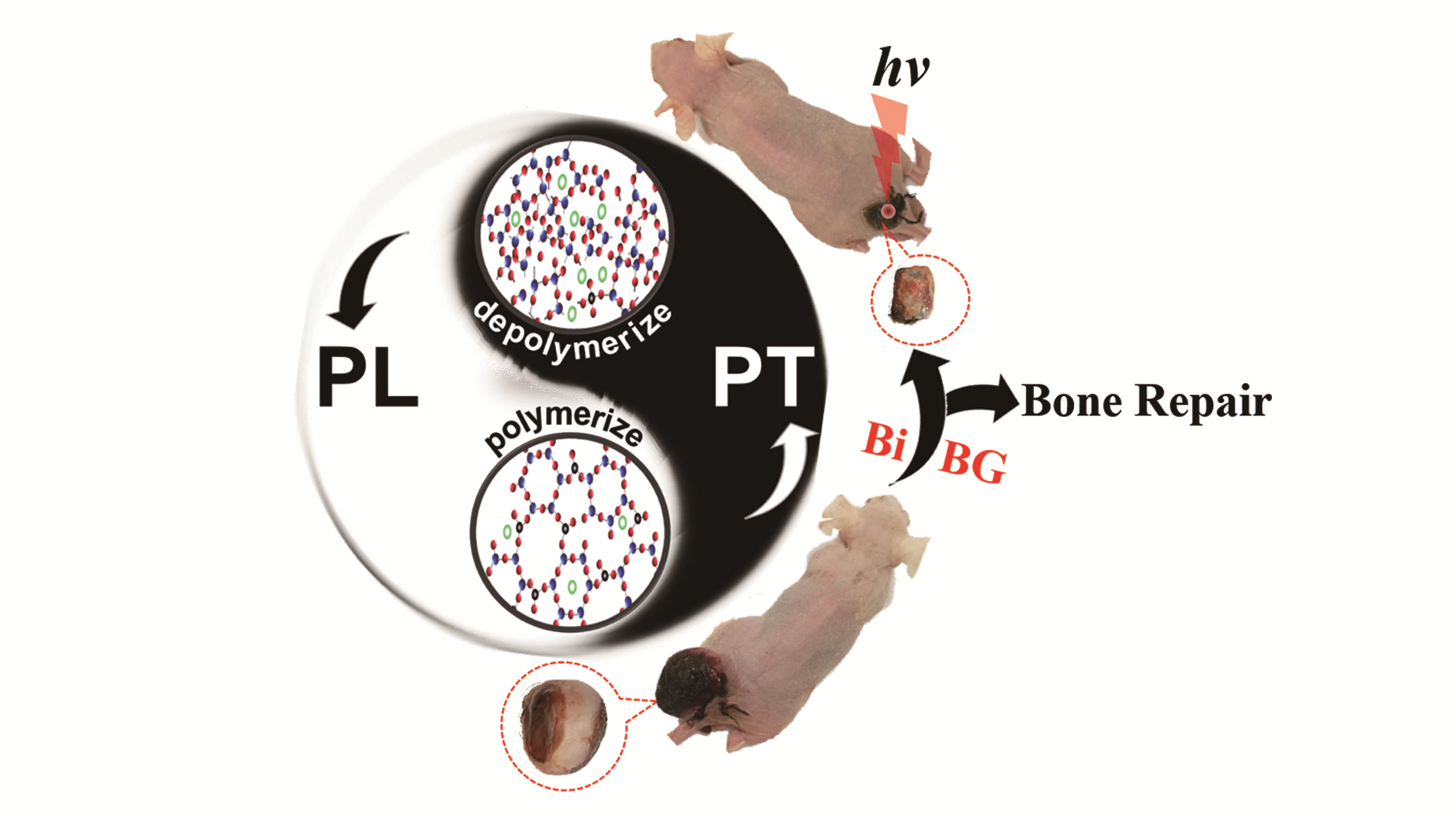


It is very challenging to control over radiation (PL) and non-radiation (PT) processes once lights are absorbed by an optical material. Here, the depolymerization of Bi glass can suppress efficiently the PL and therefore promote the PT, thus, enable the applications such as PT therapy for tumor cells or bone regeneration at the same time.
